# Performance comparison of different medical image fusion algorithms for clinical glioma grade classification with advanced magnetic resonance imaging (MRI)

**DOI:** 10.1038/s41598-023-43874-5

**Published:** 2023-10-17

**Authors:** Amir Khorasani, Nasim Dadashi serej, Milad jalilian, Azin Shayganfar, Mohamad Bagher Tavakoli

**Affiliations:** 1 Department of Medical Physics, School of Medicine, Isfahan University of Medical Sciences, Isfahan, 81746-73461 Iran; 2 Medical Image and Signal Processing Research Center, School of Advanced Technologies in Medicine, Isfahan University of Medical Sciences, Isfahan, Iran; 3 School of computing and engineering, Univesity of West London, London, UK; 4 Department of Neuroscience and Addiction Studies, School of Advanced Technologies in Medicine, Tehran University of Medical Sciences, Tehran, Iran; 5 Department of Radiology, School of Medicine, Isfahan University of Medical Sciences, Isfahan, Iran

**Keywords:** Cancer imaging, Head and neck cancer, Image processing, Machine learning

## Abstract

Non-invasive glioma grade classification is an exciting area in neuroimaging. The primary purpose of this study is to investigate the performance of different medical image fusion algorithms for glioma grading purposes by fusing advanced Magnetic Resonance Imaging (MRI) images. Ninety-six subjects underwent an Apparent diffusion coefficient (ADC) map and Susceptibility-weighted imaging (SWI) MRI scan. After preprocessing, the different medical image fusion methods used to fuse ADC maps and SWI were Principal Component Analysis (PCA), Structure-Aware, Discrete Cosine Harmonic Wavelet Transform (DCHWT), Deep-Convolutional Neural network (DNN), Dual-Discriminator conditional generative adversarial network (DDcGAN), and Laplacian Re-Decomposition (LRD). The Entropy, standard deviation (STD), peak signal-to-noise ratio (PSNR), structural similarity index measure (SSIM), and Relative Signal Contrast (RSC) were calculated for qualitative and quantitative analysis. We found high fused image quality with LRD and DDcGAN methods. Further quantitative analysis showed that RSCs in fused images in Low-Grade glioma (LGG) were significantly higher than RSCs in High-Grade glioma (HGG) with PCA, DCHWT, LRD, and DDcGAN. The Receiver Operating Characteristic (ROC) curve test highlighted that LRD and DDcGAN have the highest performance for glioma grade classification. Our work suggests using the DDcGAN and LRD networks for glioma grade classification by fusing ADC maps and SWI images.

## Introduction

Glioma is a brain tumor originating from glial cells in the brain and spinal cord. Every year, almost 100,000 people worldwide are diagnosed with glioma^1^. Glioma is recognized as a serious, worldwide public health concern because of substantial mortality and morbidity^1^. According to the tumor aggressiveness and molecular marker^2^, the World Health Organization (WHO) classified glioma grade into Low-Grade glioma (LGG) and High-Grade glioma (HGG)^3^. Glioma grade plays an essential role in managing and treating glioma tumors.

The current clinical method and the gold standard for glioma grade detection are based on histopathological findings from tumor tissue sampling. Despite its long clinical success, tissue sampling has some problems in use, such as invasiveness, time consumption, sampling error, etc.^4–6^.

In recent years, there has been an increasing interest in using medical imaging modalities for glioma grade detection as a non-invasive and fast method for glioma grading. Several attempts have been made to use the magnetic resonance imaging (MRI) modality for glioma grading as a non-invasive and fast method. Many studies have been published on the application of different MRI image weights, such as diffusion-weighted imaging (DWI)^6–12^, susceptibility-weighted imaging (SWI)^4,13–17^, perfusion-weighted imaging (PWI)^18–20^, and magnetic resonance spectroscopy (MRS)^21–23^ for glioma grade classification. A fundamental problem with much of the literature on using single MRI image weights for glioma grading is the low accuracy of these methods. It is well known that combining data from different sources can increase the glioma grade classification accuracy. Several attempts have been made to utilize simple binary logistic regression models to increase the glioma grading accuracy by ‘image data’ combination^4,24,25^. The main weakness of this method is that they offer no visible and tangible results. Recent developments in image fusion methods have led to introduce algorithms for fusing two different images to create one image that contains the information of the source images, which will be much more helpful for physicians in the clinic.

In recent years, there has been an increasing interest in introducing different image fusion methods, and researchers have proposed different methods such as generative adversarial network (GAN)^26^, Laplacian Re-Decomposition (LRD)^27^, Principal Component Analysis (PCA)^28^, Structure-Aware^29^, Discrete Cosine Harmonic Wavelet Transform (DCHWT)^30^, Deep-Convolutional Neural network (DNN)^31^, Dual-Discriminator conditional generative adversarial network (DDcGAN)^32^, etc. Some preliminary works^5,33^ were carried out recently to use medical image fusion methods with MRI images for glioma grade classification purposes. It has now been demonstrated that the accuracy of glioma grade classification can be improved with the fusion of different MRI image weights^5,33^. Recently investigators have examined the effect of different MRI image weights fusion on the accuracy of glioma grade classification. They suggest that SWI and ADC map fusion have the highest performance for glioma grade classification^33^. For this reason, we will also use SWI and ADC maps in the present study.

The clinical performance of various fusion methods in relation to glioma grading, particularly when incorporating different MRI image weights, remains a topic that lacks clarity. Therefore, the primary objective of our study was to address this knowledge gap by thoroughly investigating the clinical performance of different image fusion techniques for glioma grade classification. Specifically, we focused on evaluating the effectiveness of fusion methods such as DDcGAN, LRD, PCA, DCHWT, Structure-Aware, and DNN in accurately classifying glioma grades. It is worth noting that, to the best of our knowledge, no previous study has delved into the clinical performance of different medical image fusion methods specifically for glioma grade classification. Consequently, our research takes a pioneering approach in this area. Considering the novelty and importance of this study, we aimed to fuse the SWI and ADC maps of glioma using various fusion methods. This fusion process was carried out to classify LGG and HGG. Subsequently, we meticulously compared these fusion methods' outcomes by employing image quality assessments and quantitative parameters for Glioma grade classification.

## Results

Ninety-six patients were recruited for this study according to our inclusion criteria (49 male, 47 female with [36 84] age range). The patients were divided into the HGGs and LGGs based on their histopathological results. Just over half the samples (59.3%) were HGGs. No significant differences were found in sex and age between HGGs and LGGs.

**Figure 1:**
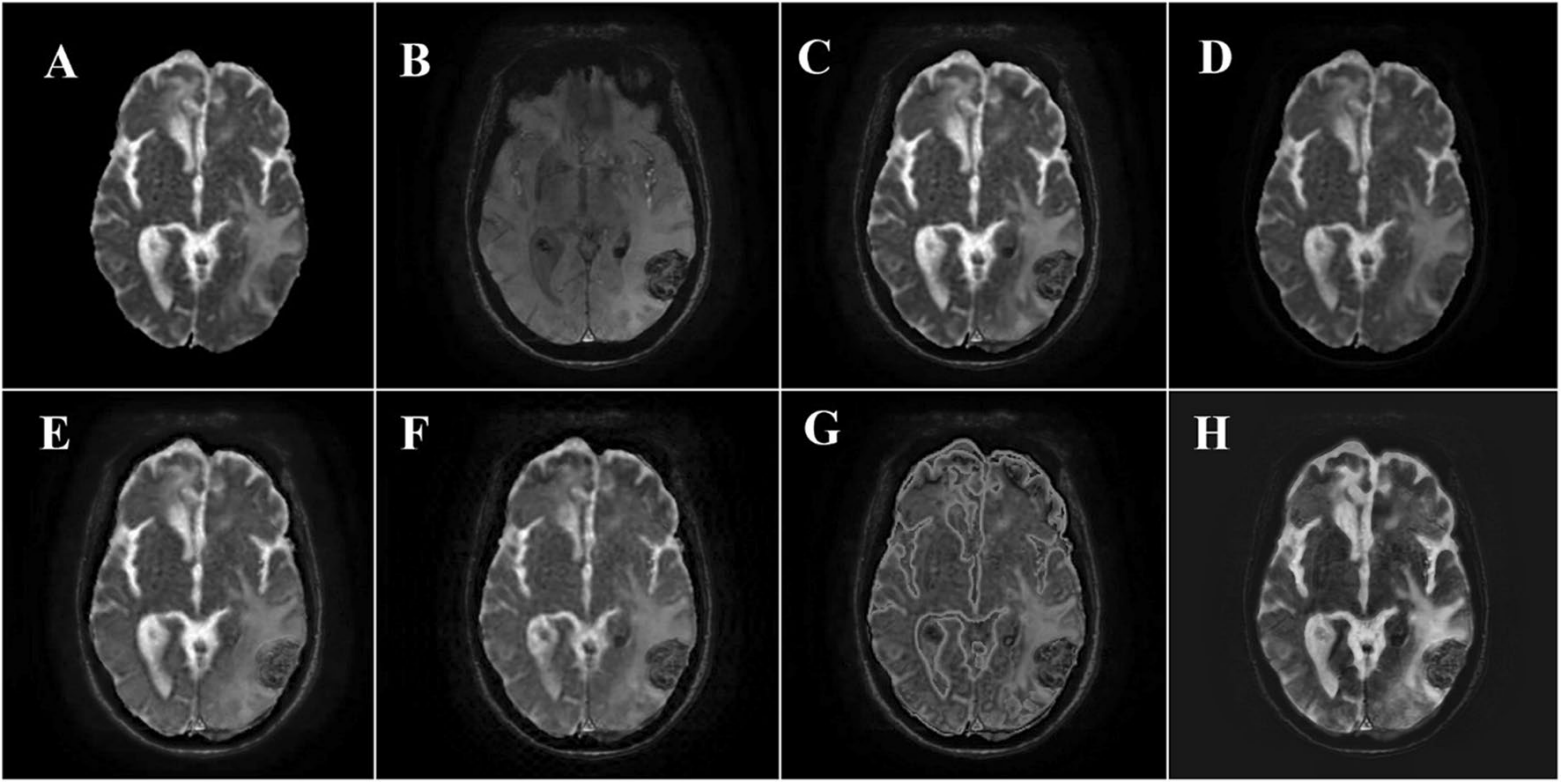
Source images and the result of different fusion methods of a 67-man with HGG. (**A**) ADC map. (**B**) SWI image. (**C**) Fused image with Structure-Aware method. (**D**) Fused image with PCA method. (**E**) Fused image with LRD method. (**F**) Fused image with DCHWT method. (**G**) Fused image with DNN method. (**H**) Fused image with DDcGAN method.

**Figure 2:**
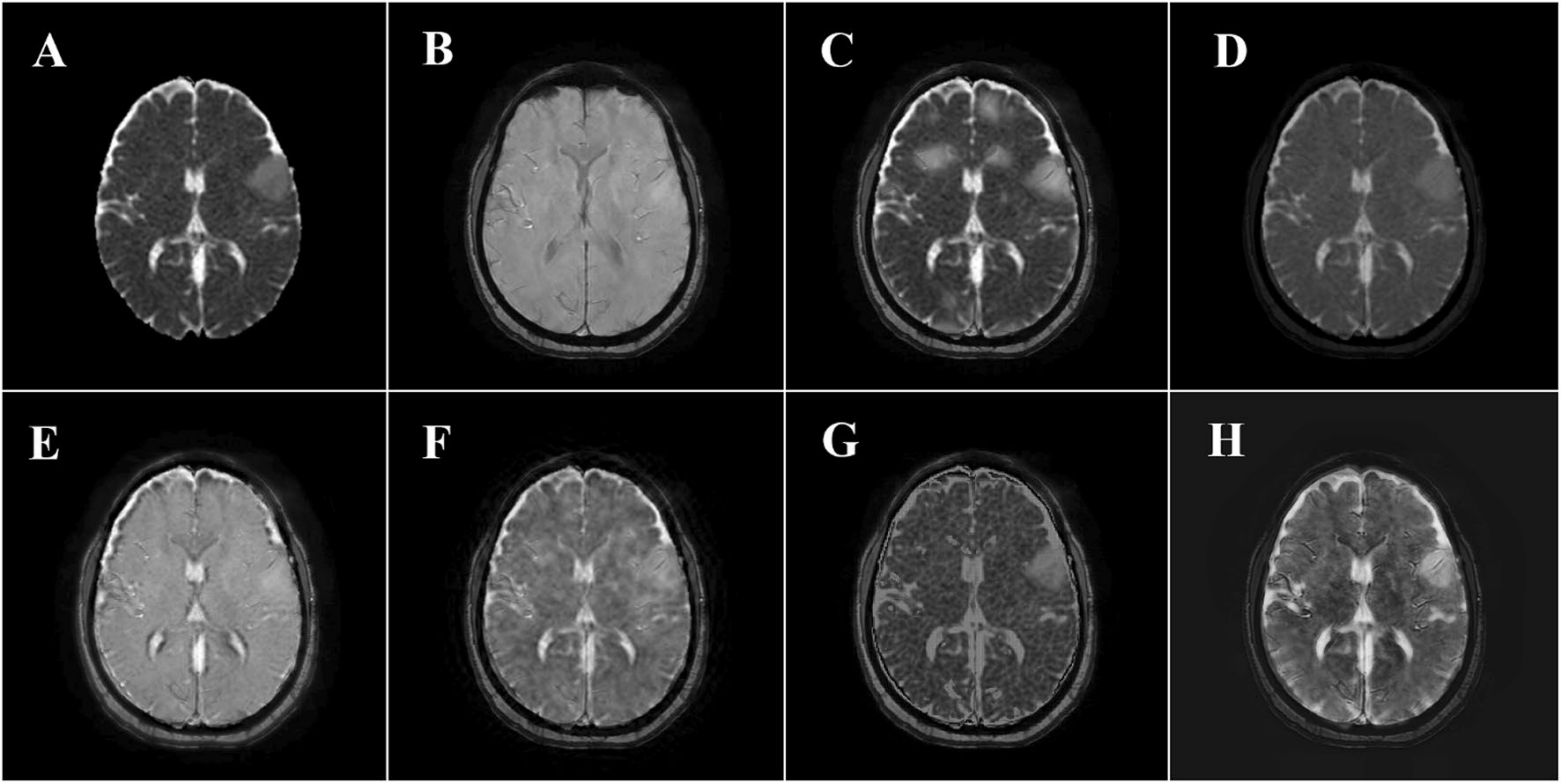
Source images and the result of different fusion methods of a 58 woman with LGG. (**A**) ADC map. (**B**) SWI image. (**C**) Fused image with Structure-Aware method. (**D**) Fused image with PCA method. (**E**) Fused image with LRD method. (**F**) Fused image with DCHWT method. (**G**) Fused image with DNN method. (**H**) Fused image with DDcGAN method.

Figures 1 and 2 show the results obtained from the different fusion methods. Figures 1 and 2 provide the fused ADC map and SWI image of an HGG and LGG patient, respectively, among the six fusion methods.

**Figure 3:**
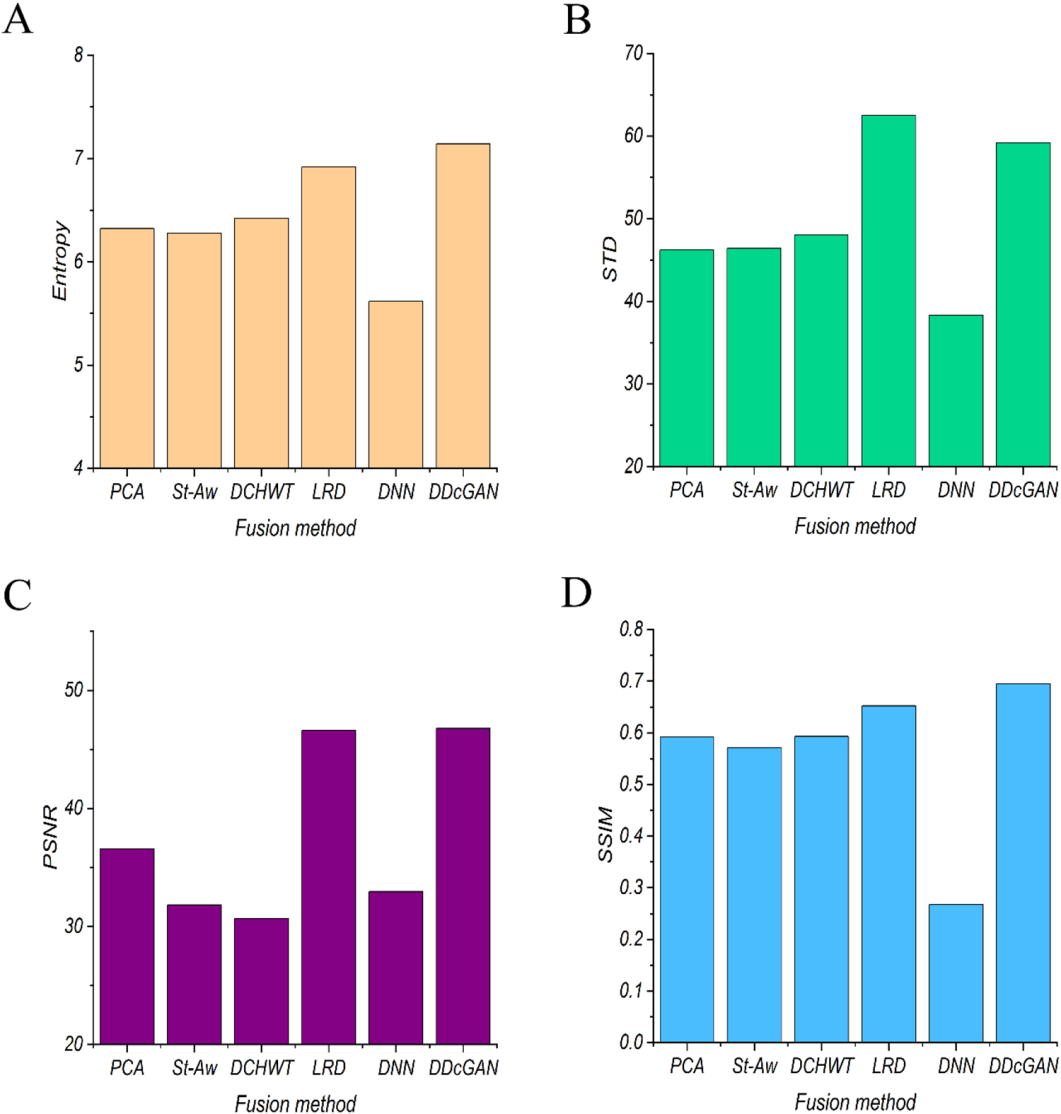
Comparative analysis of different fusion methods with image quality parameters (**A**) average Entropy, (**B**) average STD, (**C**) average PSNR, and (**D**) average SSIM in different fusion methods for fusing ADC maps and SWI images.

Table 1 and Fig. 3 show the results of comparative analysis and image quality parameters of the fused image with different fusion methods. As shown in Table 1 and Fig. 3, the results indicate that the fused images with LRD and DDcGAN significantly have the highest image quality compared with other fused methods. The average STD and entropy of the original ADC maps were calculated to be 47.02 and 5.094, respectively. For the SWI images, these values were 45.16 and 5.968, respectively.

**Table 1:** Average of Entropy, STD, PSNR, and SSIM of fused ADC map and SWI MRI image weights with different fusion methods.

Fusion method	Entropy	STD	PSNR	SSIM	Runtime (s)
PCA	6.321	46.23	36.565	0.592	0.064
Structure-Aware	6.279	46.38	31.820	0.571	1.162
DCHWT	6.421	48.06	30.670	0.593	4.075
LRD	6.921	**62.48**	46.620	0.652	982.12
DNN	5.621	38.34	32.960	0.267	0.95
DDcGAN	**7.140**	59.19	**46.780**	**0.695**	1.27

Highest values are in bold.

**Figure 4:**
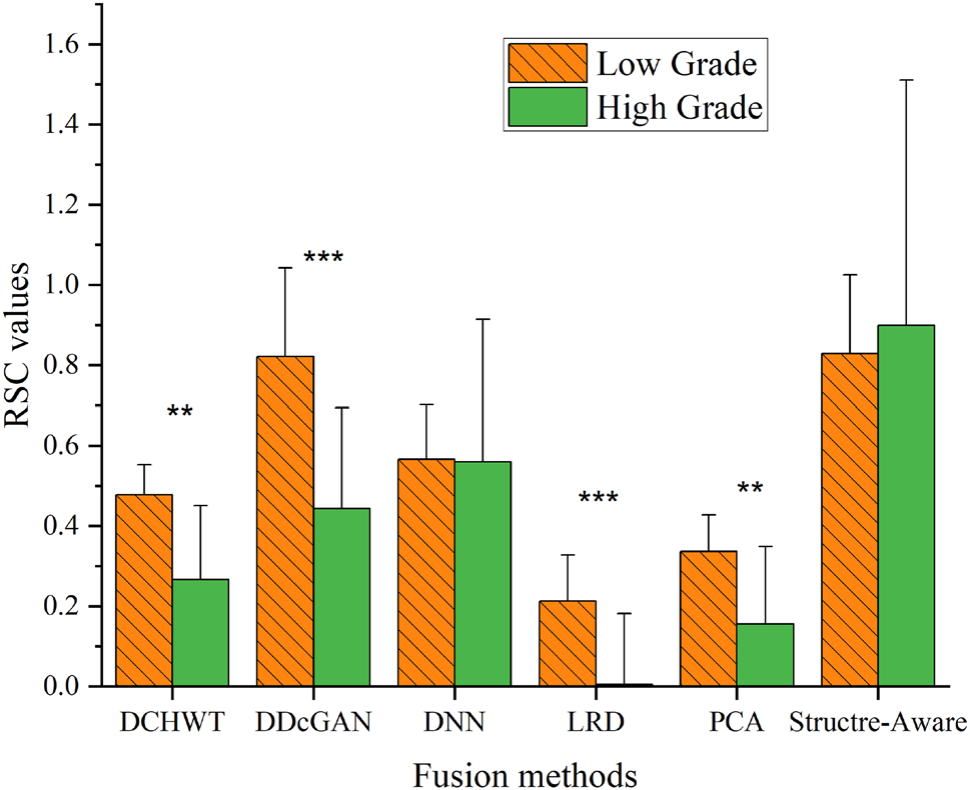
Average RSCs in LGGs and HGGs in fused ADC maps and SWI images in different fusion methods. Values are in Average ± standard deviation. ** *P* Value < 0.05, *** *P* Value < 0.01.

To evaluate the clinical performance of different fusion methods, the average RSC of fused ADC map and SWI of LGGs and HGGs were compared in different fusion methods. From the data in Fig. 4, there were no significant differences in RSCs of fused images between LGGs and HGGs with Structure-Aware and DNN fusion methods. Further analysis showed that RSCs in LGGs are significantly higher than RSCs in HGGs in fused images with PCA, DCHWT, LRD, and DDcGAN fusion methods.

**Figure 5:**
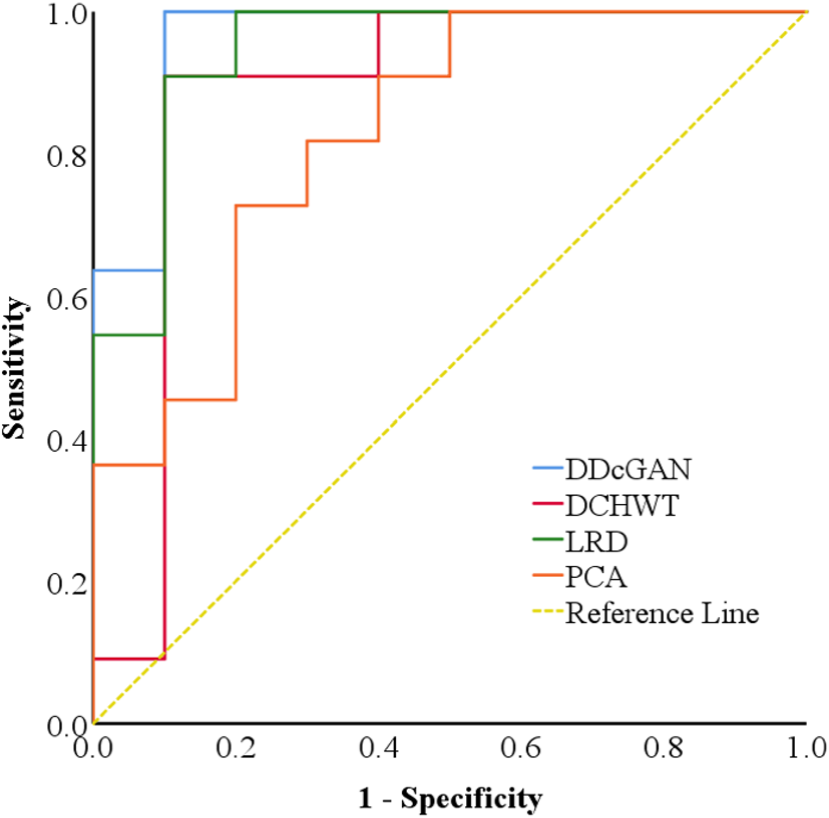
ROC curve of different fusion methods for glioma grade classification on fused ADC maps and SWI images.

ROC analysis was used to analyze the performance of different fusion methods for glioma grade quantitative classification on fused ADC maps and SWI images. Table 2 and Fig. 5 present the summary ROC analysis for glioma grade classification in ADC map and SWI fused images with different fusion methods. Interestingly, the LRD and DDcGAN have the highest performance for glioma grade classification on fused ADC maps and SWI images.

**Table 2:** Parameters of ROC curve analysis for quantitative glioma grade classification on fused ADC map and SWI images with different fusion methods.

Fusion method	Cut-off Value	AUC	Sensitivity	Specificity
PCA	0.1151	0.827	0.818	0.720
DCHWT	0.4087	0.882	0.909	0.812
LRD	0.1923	**0.945**	**0.916**	**0.843**
DDcGAN	0.6197	**0.956**	**0.975**	**0.918**

Highest values are in bold.

## Discussion

As mentioned in the literature review, glioma grade classification with different MRI images are very interesting. Prior studies that have noted the importance of data combination used simple logistic regression of MRI image data to increase the glioma grade classification accuracy^4,24,25^. A large number of image fusion methods have been reported in the literature. On the other hand, although the development of medical image fusion is exciting and popular, its clinical application for glioma grade classification has received little attention. This study assessed the importance of different image fusion methods in clinical glioma grade classification and image quality. The present study was designed to determine the clinical performance of different medical image fusion methods such as PCA, Structure-Aware, DCHWT, DNN, DDcGAN, and LRD for glioma grading with fusing ADC maps and SWI images.

The current study compared the quality of fused images with Entropy, STD, PSNR, and SSIM factors as the performance metrics. Interestingly, comparisons on image quality metrics demonstrated that the DDcGAN and LRD algorithms identify the most valuable image data and keep approximately the most considerable amount of information from the source images in the fused image. These results match those observed in earlier studies. As proposed by^32^ and^27^, the evidence we found points to the high performance of the DDcGAN and LRD fusion method compared with other methods. Although DDcGAN and LRD have the same performance for ADC map and SWI image fusion, the average runtime of DDcGAN on the testing image pairs is significantly lower than LRD runtime. Therefore, it seems that we can use DDcGAN for the ADC map and SWI image fusion with adequate fused image quality. What is surprising is that, DNN has the lowest fused image quality and performance for ADC map and SWI image fusion. A possible explanation for these results may be our limited image data set for DNN training. Further work is required to train DNN with much more image.

The quantitative analysis did not show any RSCs significant difference in the fused image with DNN and Structure-Aware algorithm between LGGs and HGGs. Fused images with PCA, DCHWT, LRD, and DDcGAN algorithms provided the significant RSCs difference between LGGs and HGGs. There is a consensus among studies that used ADC maps and SWI images for glioma grade classification^4,9–13,16,17,23^. Interestingly, in fused images, RSCs in LGGs are significantly higher than HGGs with PCA, DCHWT, LRD, and DDcGAN algorithms. These values correlate satisfactorily with previous studies and further support the role of tumor cellularity and micro bleeding in HGGs^4,5,33^. In accordance with the present results, previous studies have demonstrated that tumor cellularity increased with glioma grade, and HGGs have lower ADC values in DWI images than LGGs^34–36^. The findings observed in SWI images mirror those of the previous studies that have examined the SWI image performance for glioma grading^13,37^. The findings of previous studies showed, in HGGs, average pixel intensities of SWI images were lower than LGGs.

The most remarkable result from ROC analysis is that the DDcGAN and LRD fusion algorithms achieve the best performance in the glioma grade classification with ADC map and SWI fusion. It seems possible that these results are due to the advantage of GAN networks in feature extraction in the DDcGAN algorithm and well retain image structure data and fully considering the redundant and complementary data between high-frequency sub-band images in the LRD algorithm. These findings suggest that image fusion of ADC map and SWI with DDcGAN and LRD networks can be used for glioma grade classification in the clinic.

This investigation aimed to determine the clinical performance of PCA, Structure-Aware, DCHWT, DNN, DDcGAN, and LRD image fusion algorithms for glioma grade classification by fusing ADC maps and SWI images. Results indicated that the DDcGAN and LRD showed superiority over PCA, Structure-Aware, DCHWT, and DNN for glioma grade classification not only in qualitative analysis and fused image quality but also in quantitative analysis. However, the long-running time of the LRD algorithm cannot be ignored. For the first time, this study has demonstrated that we can use the DDcGAN network for glioma grade classification with high image quality and performance by fusing ADC maps and SWI images. The major limitation of this study is based on the small sample size. The current code runtime was limited by computer hardware. As you know, the duration of neural network training is inversely dependent on GPU processing power. It is recommended that future research be undertaken with powerful GPUs, CPUs, and a bigger sample size. Further research on optimizing and speeding up the LRD medical image fusion would be interesting. More research is required to determine the efficacy of different medical image fusion methods in the clinic, especially in radiation therapy for fusing MRI and computed tomography (CT) images for treatment planning. It would be interesting to assess the effectiveness of LRD and DDcGAN networks in radiation therapy treatment planning systems.

## Material and methods

### Patient

Criteria for patient selection were as follow:

1. Histopathology confirmation of the glioma.
2. No radiotherapy or chemotherapy was performed before the MRI exam.
3. High image quality without any MRI artifacts.
4. No claustrophobia to the MRI.
5. Do not have an allergy to the gadolinium-based contrast agent.
6. Do not have a pregnancy.

Before the head MRI scan, all participants provided informed consent, and the Isfahan University of medical sciences, Isfahan, Iran, research ethics committee approved the study with ID: IR.MUI.RESEARCH.REC.1400.237 and all parts of the experiment were performed in accordance with relevant guidelines and regulations.

### Imaging

All patients were scanned by a clinical 1.5T MR scanner (GE MRI signal explorer 1.5T) with conventional T_1_, T_2_, T_2_-FLAIR, T_1_ enhancement, DWI-weighted (ADC map), and SWI pulse sequences. The parameters of the pulse sequences are listed in Table 3.

**Table 3:** Parameters of Magnetic resonance imaging (MRI) pulse sequences. TR; time of repetition. TE; time of echo. FOV; field of view.

Image weights	TR (ms)	TE (ms)	FOV (mm)	b-value (s/mm^2^)	Matrix size	Thickness (mm)	Gap (mm)
ADC	5211	110	240 × 240	50–1000	256 × 256	5	5
SWI	87	47.5	240 × 240	–	256 × 256	5	5

### Preprocessing

Noise reduction and image smoothing, normalization, and registration techniques were used to enhance image quality and processing capability. Denoising, normalizing, image smoothing, and registration were performed using homemade custom code written in Python programming language.

#### Noise reduction

The noise reduction and image smoothing processes increased input image quality for medical image analysis. Noise reduction was employed since this is an essential preprocessing step in medical image analysis. This study used the Deep-Learning based model, a Convolutional autoencoder network, for image denoising purposes. Our convolutional autoencoder network for denoising proceeds in the same way as indicated in^38^. The encoding part consists of two convolutional layers and two max-pooling layers. The decoding consists of two convolutional layers and two upsampling layers, and in the last layer, we used a convolutional layer with one filter. More details are given in the original paper^38^.

#### Normalization

Ideally, image pixel values and contrast represent the imaged tissue properties. Nevertheless, image pixel values change due to noise and imaging artifacts. Normalization aims to reduce the effect of noise and imaging artifacts on the image's pixel values. We used the Removal of Artificial Voxel Effect by Linear Regression (RAVEL) method to do the normalization process. The RAVEL method is essentially the same as that used by Fortin et al.^39^.

#### Image registration

Image registration was done to align source images into one framework. So corresponding pixels of each source image show similar tissue points of the patient body. The Landmark-Based registration method is used to register source images^40^. This method was chosen because it is one of the most practical and straightforward ways to image registration^40^. An experienced team of neuroimaging experts and radiologists manually annotated anatomical landmarks in each MRI image, including Anterior and Posterior Commissures (AC-PC), corpus callosum, interhemispheric fissure, offer valuable landmarks, Ventricles, Basal Ganglia, Cerebellum, etc. based on Glioma location. A non-rigid transformation model using B-splines was selected for the alignment and transformation stage based on the detected landmarks and their correspondence^41–43^. The transformation parameters were estimated using a registration optimization algorithm to minimize the differences between corresponding landmarks and achieve accurate alignment^44^. The source image was then resampled and transformed using the B-spline interpolation^41,42^ techniques to map each voxel to its new position in the target image domain. Thus, the images are registered and ready for fusion.

### Fusion

The current study uses various medical image fusion methods to compare the glioma grading performance of these methods with the Dual-Discriminator conditional generative adversarial network (DDcGAN)^32^ and Laplacian Re-Decomposition (LRD)^27^ methods. This method included Principal Component Analysis (PCA), Structure-Aware, Discrete Cosine Harmonic Wavelet Transform (DCHWT), Deep-Convolutional Neural Network (DNN), DDcGAN, and LRD.

#### Dual-Discriminator conditional generative adversarial network (DDcGAN)

**Figure 6:**
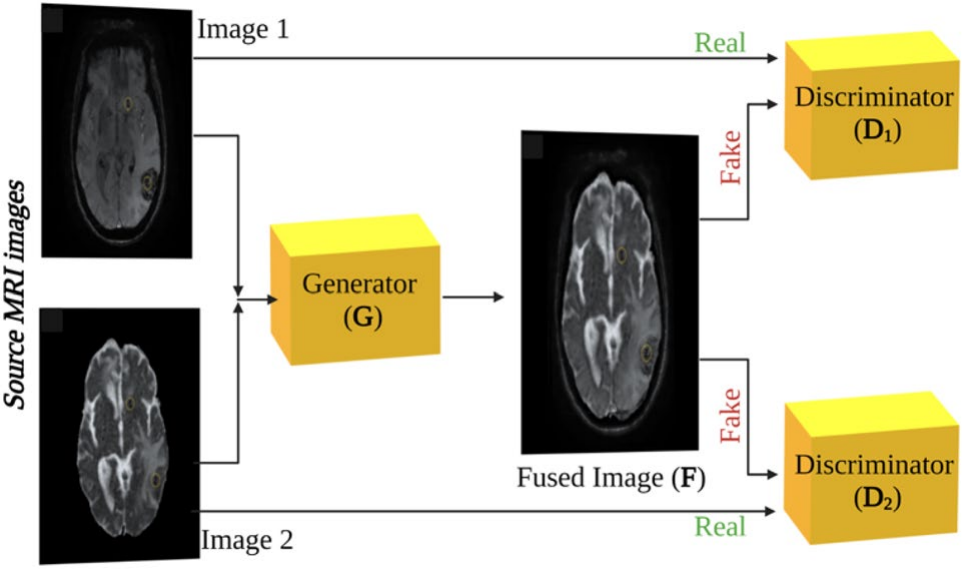
the overall DDcGAN for SWI and ADC map MRI image fusion for glioma grading.

For this study, the DDcGAN was used to fuse SWI and ADC maps for glioma grading purposes. The overall DDcGAN architecture is shown in Fig. 6. DDcGAN was created according to the procedure proposed by Ma et al.^32^. The DDcGAN goal is to learn a generator to generate fused image F to be informative and realistic enough to fool the discriminators.

**Figure 7:**
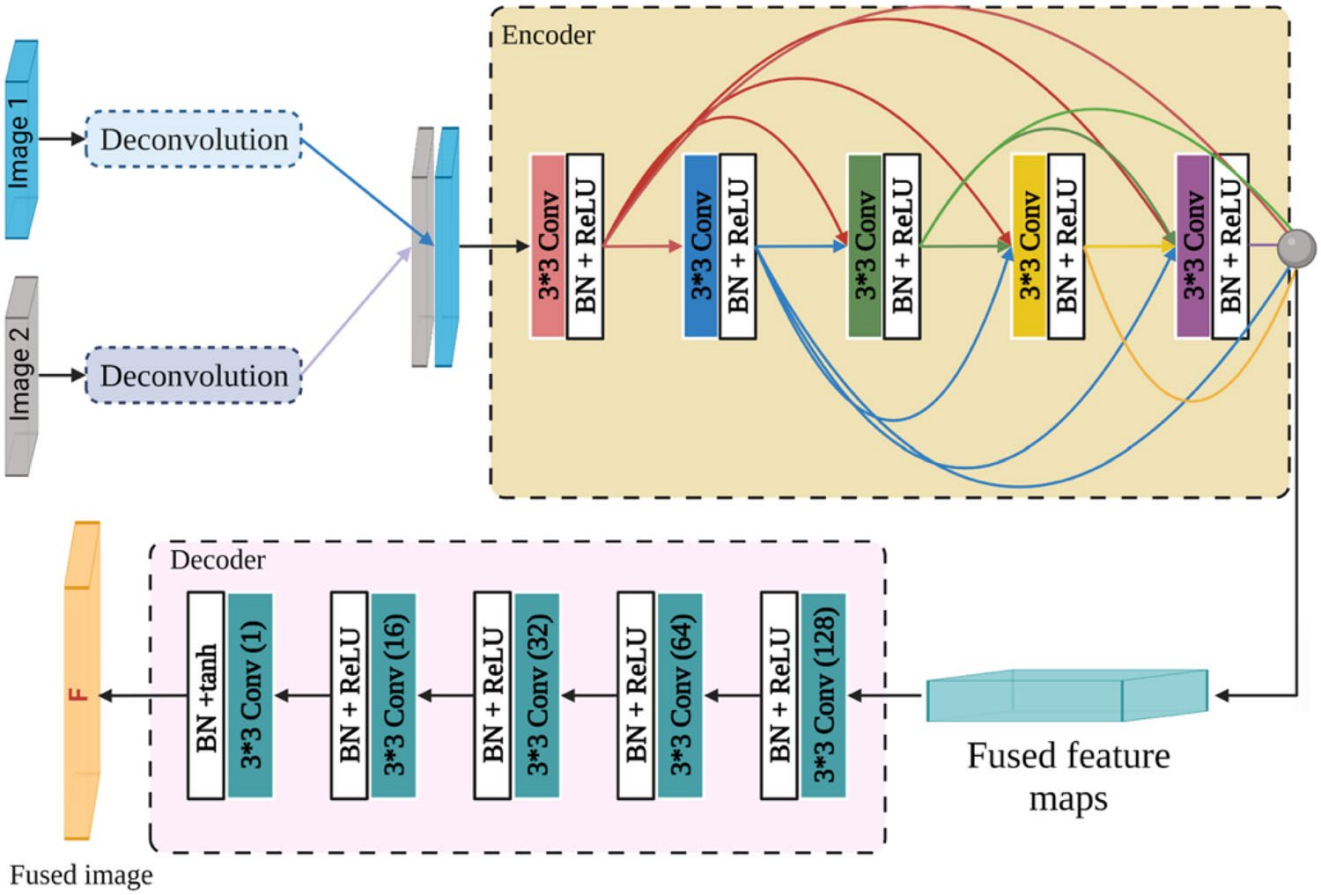
the generator architecture, including encoder and decoder networks. 3*3 is the filter size, Conv (n) is the convolution layer with n filter number, BN is batch normalization, and ReLU and tanh are activation functions.

The generator architecture is presented in Fig. 7 and consists of two deconvolution layers, an encoder section, and a decoder section. Deconvolution layers obtain the feature map of the source images. These feature maps are concatenated and used as an input image of the encoder network.

The encoder network consists of five convolutional layers. Each convolutional layer consists of a 3 × 3 filter size with stride 1, batch normalization, and ReLU activation function. Short direct connection and DenseNet^45^ are used between each layer and all layers in a feed-forward fashion. The fused feature maps are produced as the output of the encoder network. Following this, the encoder output is fed to the decoder network to generate and reconstruct fused images.

The decoder part is also made of five convolutional layers, as shown in Fig. 7. The decoder layers are very similar to the encoder layers, except that tanh is used as the activation function of the last layer of the decoder part.

**Figure 8:**
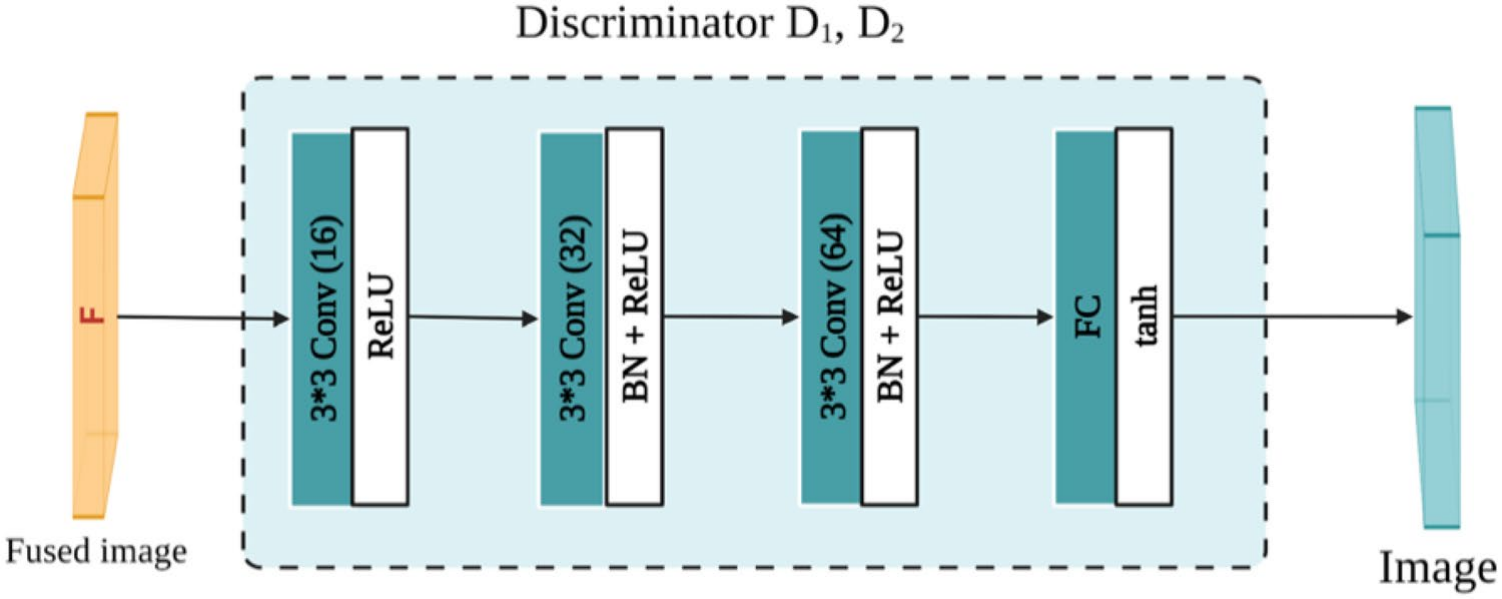
the Discriminator architecture. 3 × 3 is the filter size, Conv (n) is the convolution layer with n filter number, BN is batch normalization, ReLU and tanh are activation functions, and FC is fully connected.

The design of DDcGAN architecture was based on the use of two discriminators. The architecture of the discriminators D_1_ and D_2_ are similar, as shown in Fig. 8. D_1_ and D_2_ are made of three convolutional and one fully connected layer. Convolutional layers consist of a 3 × 3 filter size with different filter numbers, batch normalization, and ReLU activation function. In the fully connected layer, the tanh activation function was used. The DDcGAN architecture we used is detailed in^32^.

#### Laplacian re-decomposition (LRD)

**Figure 9:**
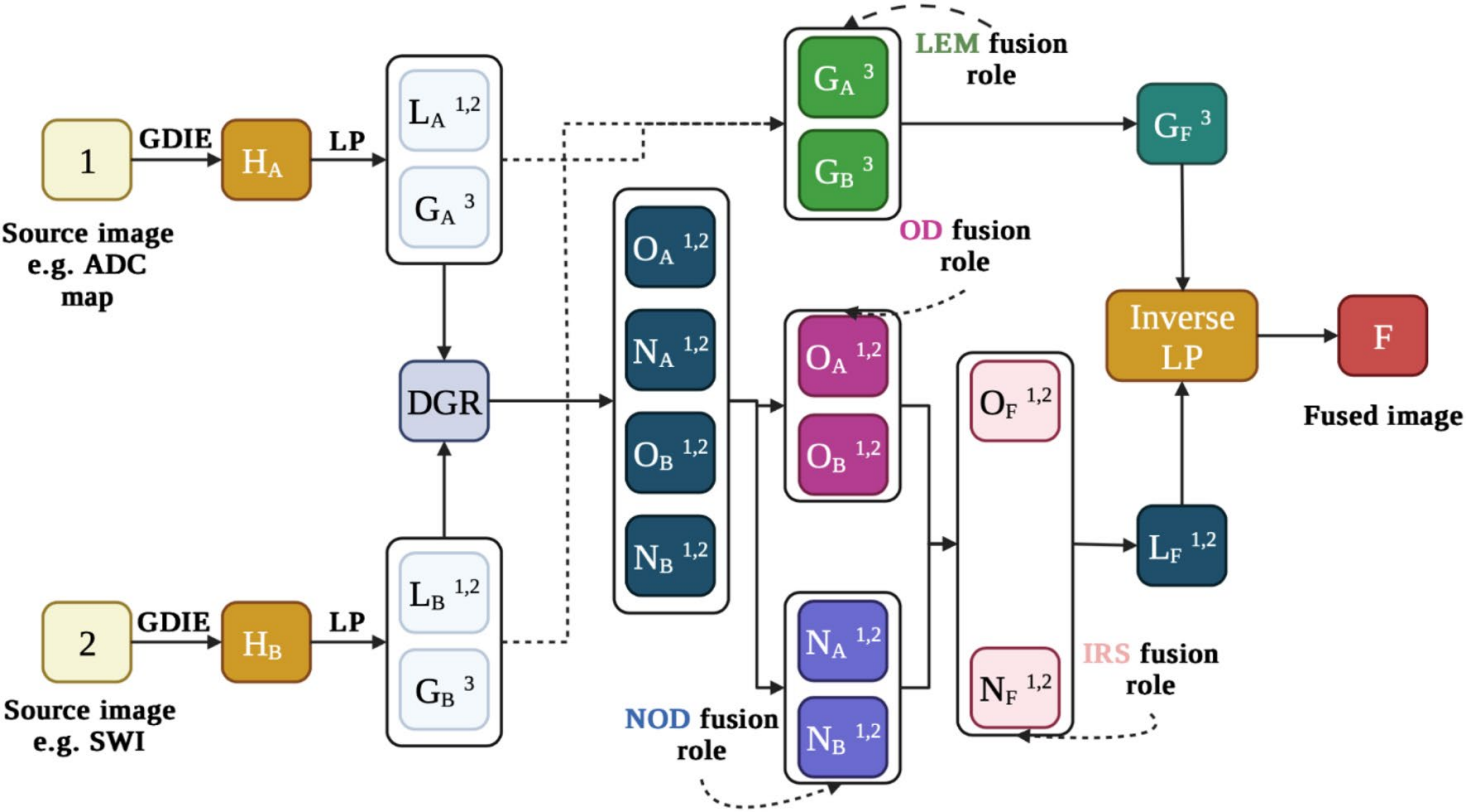
The overall LRD fusion method for medical image fusion. In this algorithm, 1 and 2: source images, H_A_ and H_B_: enhancement images, L_A_ and L_B_: High-frequency sub-band images, G_A_ and G_B_: Low-frequency sub-band images, O_A_ and O_B_: overlapping domain images, N_A_ and N_B_: Non-overlapping domain images, O_F_: overlapping domain fusion images, N_F_: Non-overlapping domain fusion images, G_F_: low-frequency sub-band fusion images, L_F_: high-frequency sub-band fusion images, F: fused image.

The several steps of LRD fusion methods are in Fig. 9. Briefly, In the LRD methods, each source image's enhancement images (HA and HB) are produced by Gradient Domain Image Enhancement (GDIE). Following this, by using Laplacian pyramid transform (LP) from H_A_ and H_B_ images, High-frequency (L_A_ and L_B_) and Low-frequency (G_A_ and G_B_) sub-band images were created. The Overlapping domain (O_A_ and O_B_) and non-overlapping domain (N_A_ and N_B_) images were created with the Decision Graph Re-decomposition (DGR). In the last stage of the LRD fusion method, with different fusion rules such as local energy maximum (LEM), overlapping domain (OD), non-overlapping domain (NOD), inverse re-decomposition scheme (IRS), and inversed LP, the fused image was reconstructed. More details of this medical fusion algorithm are in^27^. We used the MATLAB software package for writing and running LRD fusion code.

#### PCA, Structure-Aware, DCHWT, and DNN medical image fusion methods

**Figure 10:**
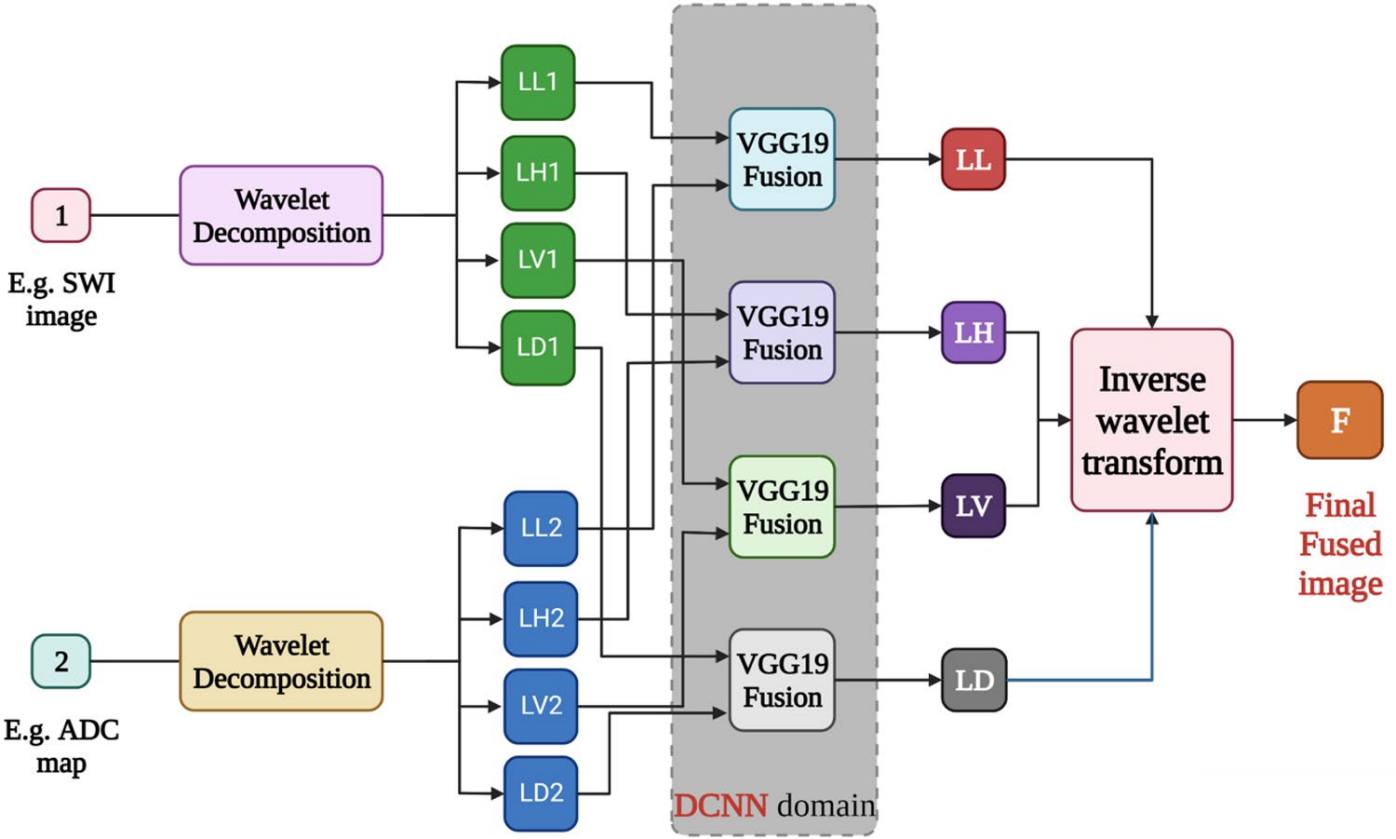
The overall DNN fusion method for medical image fusion.

In fact, to compare the DDcGAN and LRD performance for glioma grading, we used different image fusion methods such as PCA, Structure-Aware, DCHWT, and DNN. For example, the general structure of the DNN is shown in Fig. 10. In this section, our goal is not to describe the details of each fusion method. Details can be found in the original paper on the proposed fusion methods^27–32^.

### Performance analysis of fusion methods

Most recent studies have measured fused image visual quality in different accepted standard ways^46^. These standard factors are Entropy, standard deviation (STD), peak signal-to-noise ratio (PSNR), and structural similarity index measure (SSIM)^46^.

#### Entropy

Image entropy is a metric used to quantify the amount of information or randomness present in an image. It measures the average uncertainty or disorder within the image. In other words, image entropy provides a measure of the complexity or variability of pixel values in an image. Entropy is calculated based on the probability distribution of pixel intensities within the image. A higher entropy value indicates more significant variability and randomness in the pixel values, while a lower entropy value suggests a more uniform or predictable distribution. Entropy is calculated as follows^46^:

1 $$entropy = - \mathop \sum \limits_{L = 0}^{L - 1} P_{i} \times \log_{2} P_{i}$$

where L is the gray level number in the image, P is the probability density function for the gray level of *i*. The range of image entropy depends on the number of intensity levels used for quantization. If an image is represented using 'L' discrete intensity levels (e.g., 8-bit grayscale images with 256 levels), then the entropy values typically range from 0 to log2(L). In an 8-bit grayscale image (L = 256), the entropy values can range from 0 to 8. Entropy is proportional to the amount of fused image information in fused images.

#### STD

Image STD is a statistical measure that quantifies the amount of variation or dispersion in pixel intensities within an image. It provides information about the spread of pixel values around the mean intensity. STD is used to measure the image contrast of the fused image. The higher STD values represent more helpful information and high contrast of the fused image. STD calculated as^46^:

2 $$STD = \sqrt {\frac{{\mathop \sum \nolimits_{i = 1}^{M} \mathop \sum \nolimits_{j = 1}^{N} \left( {f\left( {i,j} \right) - \mu } \right)^{2} }}{M \times N}}$$

where M and N are the length and width of the fused image, f(i,j), μ is the average gray value of the fused image.

#### PSNR

PSNR is a quantitative value based on mean square error. The higher PSNR value in the fused image is better and calculated as follows^46^:

3 $$PSNR = 10 \times \log_{10} \left( {\frac{{L^{2} }}{{RMSE^{2} }}} \right)$$

where L is the maximum gray level value in the fused image, and RMSE is the mean square error and calculated with the following equation^46^:

4 $$RMSE = \sqrt {\frac{{\mathop \sum \nolimits_{m = 1}^{M} \mathop \sum \nolimits_{n = 1}^{N} \left[ {A\left( {m,n} \right) - F\left( {m,n} \right)} \right]^{2} }}{M \times N}}$$

A(m, n) and F(m, n) represent the intensity value of the source and fused images, respectively. M and N are the length and width of images.

#### SSIM

SSIM is a factor that measures the structural similarity between the source image and the fused image. SSIM values between 0 and 1 and higher SSIM values indicate a higher structural similarity between the two images. SSIM calculated as^46^:

5 $$SSIM_{{\left( {A,B,F} \right)}} = \frac{{SSIM_{{\left( {A,F} \right)}} + SSIM_{{\left( {B,F} \right)}} }}{2}$$

6 $$SSIM_{{\left( {A,F} \right)}} = \frac{{\left( {2\mu_{A} \mu_{F} + C_{1} } \right)\left( {2\sigma_{AF} + C_{2} } \right)}}{{\left( {\mu_{A}^{2} + \mu_{F}^{2} + C_{1} } \right)\left( {\sigma_{A}^{2} + \sigma_{F}^{2} + C_{2} } \right)}}$$

7 $$SSIM_{{\left( {B,F} \right)}} = \frac{{\left( {2\mu_{B} \mu_{F} + C_{1} } \right)\left( {2\sigma_{BF} + C_{2} } \right)}}{{\left( {\mu_{B}^{2} + \mu_{F}^{2} + C_{1} } \right)\left( {\sigma_{B}^{2} + \sigma_{F}^{2} + C_{2} } \right)}}$$

A and B are source images, and F is the fused image. In these equations, µ is the average value, and σ is the variance of source and fused images. C_1_ and C_2_ are small constants added for numerical stability.

### Quantitative evaluation

The study uses quantitative analysis to gain insights into the clinical application of different fusion methods for glioma grading with MRI images. For the quantitative analysis, for each patient, the image slice with the largest tumor size and the highest quality was selected for data evaluation by two radiologists. To determine the Relative Signal Contrast (RSC) values, the mean signal intensity of circular regions of interest (ROI) in each image was calculated. Radiologists manually drew the ROIs with ImageJ software packages. Three ROIs were sampled and averaged for each region to improve the comparison's power. Then, RSC was calculated by the following equation^5,33^:

8 $$RSC_{ROI} = \frac{{\mu_{ROI} - \mu_{WM} }}{{\mu_{WM} }}$$

${\mu }_{ROI}$ Is the mean signal intensity of the active tumor region, and ${\mu }_{WM}$ Is the mean signal intensity of the white matter in each picture. RSCs in fused images of LGGs and HGGs with different fusion methods were calculated and compared with each other for glioma grading performance.

### Statistical analysis

Data management and analysis were performed using SPSS 26.0 software (IBM Corp. Armonk, NY, USA). Kolmogorov–Smirnov test can be used to assess the normality distribution of the data. We could compare RSCs between LGGs and HGGs using the two-tailed unpaired student's T-Test. We used the Exact Fisher test to evaluate the sex and age relationship with the glioma grade. The *P* values of less than 0.05 were indicated to be statistically significant. The significant RSCs for the LGGs and HGGs were subjected to receiver operating characteristic (ROC) curve analysis to determine the cut-off values, the area under the curve (AUC), sensitivity, and specificity for glioma grade classification in fused MRI images with different fusion methods. The area under the curve (AUC) was calculated with the cut-off value set as the maximum Youden index.

## Data Availability

The datasets used and analyzed during the current study are available from the corresponding author upon reasonable request.
